# Re-introduction of an extinct population of *Pulsatilla patens* using different propagation techniques

**DOI:** 10.1038/s41598-022-18397-0

**Published:** 2022-08-22

**Authors:** Justyna Żabicka, Piotr Żabicki, Aneta Słomka, Elwira Sliwinska, Monika Jędrzejczyk-Korycińska, Teresa Nowak, Grzegorz Migdałek, Monika Kwiatkowska, Elżbieta Kuta

**Affiliations:** 1 Department of Plant Cytology and Embryology, Institute of Botany, Faculty of Biology, Jagiellonian University in Kraków, 9 Gronostajowa St., 30-387 Cracow, Poland; 2 Laboratory of Molecular Biology and Cytometry, Department of Agricultural Biotechnology, Bydgoszcz University of Science and Technology, Kaliskiego Ave. 7, 85-796 Bydgoszcz, Poland; 3 Institute of Biology, Biotechnology and Environmental Protection, Faculty of Natural Sciences, University of Silesia, 28 Jagiellońska St., 40-032 Katowice, Poland; 4 Institute of Biology, Pedagogical University of Cracow, 2 Podchorążych St., 30-084 Cracow, Poland

**Keywords:** Biological techniques, Biotechnology, Molecular biology, Plant sciences

## Abstract

The study focuses on the propagation of a rare and endangered plant species (*Pulsatilla patens*) to re-introduce an extinct population from calamine area in Southern Poland. The plants were propagated from seeds, rhizome cuttings, or regenerated in vitro from shoot tips, hypocotyls with roots or cotyledons of seedlings on Murashige & Skoog (MS) medium supplemented with 0.25 or 0.50 mg L^−1^ BAP (Benzylaminopurine) via direct and indirect organogenesis or somatic embryogenesis (SE). The most efficient micropropagation method was with shoot tips as an explant on MS + 0.25 mg L^−1^ BAP where 97% of the explants produced multiple shoots, mass SE was observed after transfer on ½ MS with 2% saccharose; 267 (35%) shoots rooted on ½ MS + 2% saccharose were acclimatized to *ex vitro* conditions. Flow cytometry revealed genome size stability of propagated plantlets. Low genetic differentiation between micropropagated plantlets and initial material was indicated by ISSR (Inter Simple Sequence Repeat) markers. Totally, 132 vigorous plantlets obtained on various pathways were introduced to the field plots in 2020; 30.33% survived the winter, and several reached the generative stage and flowered in the spring 2021. In next season (March/April 2022) the number of introduced plants decreased to 25% while the number of flowering and fruiting shoots in different clumps increased in some plots. This is the first report of successful re-introduction of the endangered *P. patens* based on micropropagation, rhizome cuttings, and seed germination.

## Introduction

### Is there a risk of extinction of the pasque-flower in its European range?

*Pulsatilla patens* (L.) Mill. (pasque-flower) is a one of the most endangered plant species in Europe, and in several regions it is threatened with extinction^1–13^. Current threats include both natural and anthropogenic factors, e.g. habitat reduction, fragmentation and eutrophication, natural succession, unsuitable forest management, forest fires, damage caused by animals, reduced seed set due to a lack of pollinators, poor seed germination, scarce vegetative propagation, hybridization with other pasque-flower species, and a lack of root-colonizing mycorrhizal fungi^1,8,14–18^. Variation in the life cycle and slow growth and development until flowering are also negative factors for population dynamics^19^. Thus, the species is included in the European Habitats Directive 92/43/EEC, Appendix II i IV, code 1477. In Poland, in the late 19th and early twentieth century the range of *P. patens* included almost the entire country. The situation drastically changed in the second half of the twentieth century, when almost all populations in western, central and southern Poland became extinct. Currently, the highest density of extant populations is reported from north-eastern Poland^11^.

### Unique biodiversity of calamine grasslands needs effective protection and restoration

*Pulsatilla patens* occurs in oligotrophic and mesotrophic habitats on sand and argillaceous clay, e.g. in grasslands and half-open forests. The species was formerly abundant at many metalliferous (Zn-Pb rich; calamine) sites in southern Poland, e.g. in Bolesław, Stary Olkusz and Jaworzno^20,21^; currently not a single plant remained in this area (monitoring in 2019). The active protection and restoration of populations in calamine grasslands was highlighted recently because metallicolous vegetation forms unique assemblages of a limited number of metallophyte species that successfully colonized these sites. These assemblages are the result of microevolutionary processes in stressful environments, and there is only a limited number of sites where they can be observed^22,23^. Metallophyte species are also unique resources that could be exploited for the development of phytoremediation including revegetation^24^. Calamine grasslands benefit from conservation programs and are considered as a threaten habitat type that is legally protected by European (European Habitats Directive 92/43/EEC, Appendix I, habitat type 6130), national or regional laws. Among sites of the Natura 2000 network, 118 are designated for this habitat type^25^.

### Techniques of multiplication and cryopreservation—the hope for maintaining the genetic pool of pasque-flower

The preservation of endangered, threatened plant species by ex situ conservation, including in vitro culture and cryopreservation, prevents losses of plant biodiversity^26^, recovers genetic diversity from preserved material, and allows transplantation of multiplied plants to botanical gardens and field sites^27–30^.

In vitro culture serves as an excellent biotechnological tool for ex situ conservation of different endangered species^31,32^, including *Pulsatilla vulgaris*^33^, and also species growing at metalliferous sites^34–36^. However, the re-introdution of in vitro propagated plants to the field sites, will be successful provided the areas are properly managed before the transplantation^22,25,37^.

The main goals of the present study was to restore an extinct calamine grassland population of the endangered *P. patens* by using plants propagated from seeds, rhizome cuttings, or micropropagated in vitro*,* and to monitor their successful acclimatization to the field conditions. Molecular markers and flow cytometry were used to assess the genetic quality of the in vitro regenerated plantlets.

## Results

### Cytological characteristic of plants

**Figure 1:**
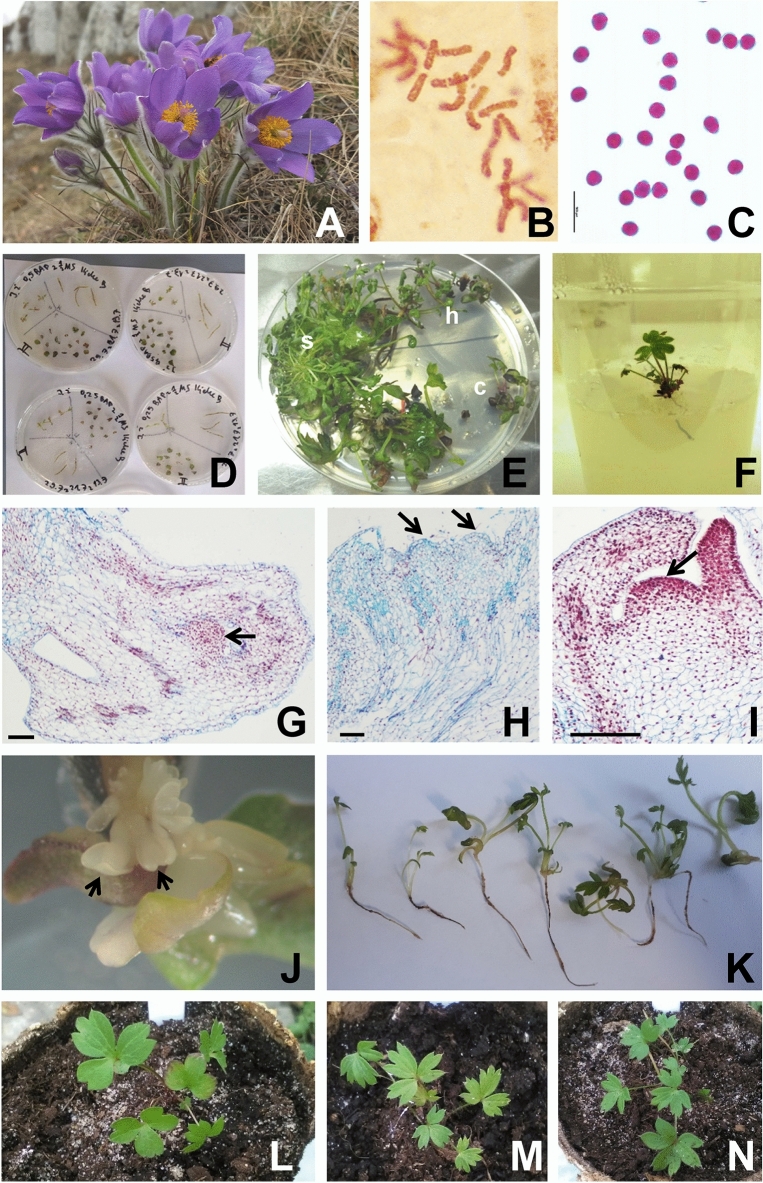
*Pulsatilla patens* micropropagation and acclimatization of regenerated plantlets. (**A**) Flowering plants in a natural stand in Łagiewniki near Busko-Zdrój, southern Poland. (**B**) Metaphase plate with 16 somatic chromosomes. (**C**) Viable (stainable) pollen grains of plants introduced to Sadowa Góra in Jaworzno. (**D**) Shoot tips, cotyledon fragments and hypocotyls with roots on induction media MS (Murashige & Skoog) + 0.25 or 0.5 mg L^−1^ BAP. (**E**) Organogenesis on the induction medium, varied response of explants, low of cotyledons (c), mass adventitious shoots formation on shoot tips (s) and hypocotyls (h). (**F**) Rooting on ½ MS (2% saccharose). (**G**, **H**) Meristematic centers (arrows) differentiated directly in the explant on induction medium. (**I**) Shoot tip (arrow), direct organogenesis. (**J**) Somatic embryos (SE) at heart stage (arrows) on explants (shoot tips) on ½ MS (2% saccharose). (**K**) Seedlings developing from SE. (**L**–**N**) Regenerated plantlets in *ex vitro* conditions in substrate, acclimatized to outdoor conditions: from somatic embryos (**L**) and from rooting adventitious shoots (**M**, **N**). (**G**–**I**) Microtome sections of explants on induction medium stained with Ehrlich's hematoxylin and alcian blue. Bar = 100 µm (**G**–**I**).

*Pulsatilla patens* from Łagiewniki near Busko-Zdrój (Fig. 1A) was diploid with somatic chromosome number 2*n* = 2*x* = 16 (Fig. 1B). High pollen (isopolar, tricolpate) viability, reaching 100% of plants from the same site as well as of flowers of introduced plants, and regular size and shape of viable pollen grains indicated regular male line development. Non-viable (green or empty), giant or dwarf pollen grains were not observed neither in plants from Łagiewniki near Busko-Zdrój nor in plants introduced to Sadowa Góra (Fig. 1C).

### Poor seed germination and plantlets development from rhizome cuttings

For seed germination tests, 1022 fruits were used (Table 1). Germination and seedlings development depended on the substrate. The highest germination (73%) was on ½ MS (Murashige & Skoog) with 2% saccharose, very low in garden soil (17%), and deacidified peat mixed with sand (18%). Five plants recovered in spring 2020 from rhizome cuttings were grown in a common garden until transplantation to Sadowa Góra in October 2020.

**Table 1:** Seed germination of *Pulsatilla patens* on different substrates.

Medium	No. of seeds used	No. of germinating seeds/developed seedlings [%]
½ MS (2% saccharose); in vitro conditions	418	305 [72.97]
Deacidified peat mixed with sand (3:1 v/v); indoor, outdoor conditions	144	26 [18.06]
Garden soil; indoor, outdoor conditions	460	80 [17.39]
Total	1022	411 [40.22]

MS—Murashige & Skoog^38^ medium.

### Organogenesis and somatic embryogenesis, rooting and acclimatization as a method of restoring population

Direct and indirect (via callus) organogenesis (adventitious shoot development) was induced in all used explants, shoot tip, cotyledon, hypocotyl + root (Fig. 1D,E) on MS medium supplemented with BAP (Benzylaminopurine) in concentrations 0.25 or 0.50 mg L^−1^. Two-way ANOVA showed significant differences between types of explants but not between the media supplemented with different concentrations of BAP (MS + 0.25 or 0.50 mg L^−1^ BAP). Interaction between type of explants and medium was also non-significant. Tukey’s HSD test *post-hoc* confirmed significant differences between hypocotyl + root on MS + 0.25 mg L^−1^ BAP and shoot tips (Table 2). Shoot tips excised from seedlings were the best explants. Altogether 91% responded for culture conditions, which was a much higher frequency compared to hypocotyl + root (64%) and cotyledon (7%). The most abundant adventitious shoot induction on one explant (several dozen, mass of shoots, uncountable) was observed on shoot tip explants on both growth media. Direct organogenesis was confirmed on histological sections, shoots developed from meristematic centers induced inside the explants (Fig. 1G–I). Rooting of adventitious shoots after transfer on ½ MS hormone-free medium (supplemented with 2% saccharose) was poor; out of 758 shoots obtained in three experiments, only 267 (35%) developed roots (Table 3, Fig. 1F). Rooting time was long and lasted up to several months without passaging the shoots on fresh medium.

**Table 2:** Organogenesis and somatic embryogenesis of *Pulsatilla patens* after several months of culture.

Medium	Explant type								
	Shoot tip			Cotyledon			Hypocotyl + root		
	No. of explants	No. of responding explants [%]	No. of SE*	No. of explants	No. of responding explants [%]	No. of SE*	No. of explants	No. of responding explants [%]	No. of SE*
MS + 0.25 mg L^−1^ BAP									
I	31	29+++ [93.55]	36	124	16+ [12.90]	12	31	20++ [64.52]	0
II	18	18 (2+, 6++, 10+++) [100]	7	67	5 (2+, 3++) [7.50]	0	19	12 (5+, 5++, 2+++) [63.20]	0
III	12	12 (3+, 3++, 6+++) [100]	mass SE	62	0	0	12	4 (3+, 1+++) [33.30]	0
Total	61	59 [96.72]^a^	43+ multiple SE	253	21 [8.30]	12	62	36 [58.06]^b^	0
MS + 0.50 mg L^−1^ BAP									
I	47	37+++ [78.72]	22	188	16++ [8.51]	3	47	34+++ [72.34]	0
II	18	18 (1+, 3++, 14+++) [100]	4	72	1 [1.39]	0	19	14 (6+, 5++, 3+++) [73.68]	0
III	11	11 (1+, 7++, 3+++) [100]	2	58	1++ [1.72]	0	13	6 (2+, 4++) [46.15]	0
Total	76	66 [86.84]^a^	28	318	17 [5.66]	3	79	54 [68.35]^ab^	0

SE—Somatic embryos converted into seedlings; intensity of organogenesis: + single/several shoots, ++ several dozen/dozen shoots, +++ multiple, uncountable shoots.

MS—Murashige & Skoog^38^ medium.

BAP—Benzylaminopurine.

*Induction of somatic embryogenesis after explant/callus transfer on hormone free medium ½ MS + 2% saccharose.

I–III—Repetitions of the experiments.

Cotyledons were excluded from the statistical test due to the great differences in results between cotyledons and the remining explants.

Two-way ANOVA showed statistical differences between type of explants (F (1, 8) = 22.298, p = 0.0015). Average values marked by the same letter do not differ significantly at p ≤ 0.05 as revealed by Tukey’s HSD *post-hoc* test.

**Table 3:** Efficiency of rooting on ½ MS (2% saccharose) and acclimatization of micropropagated shoots of *Pulsatilla patens* and plants converted from somatic embryos (SE).

Experiment	Rooting* and acclimatization to ex vitro conditions		No. of surviving plantlets (rooted shoots + SE) [%]
	No. of shoots	No. of rooted shoots [%] + SE**	
I	539	193 [35.81] + 38 SE	44 [19.05]
II	167	61 [36.53] + 12 SE	0
III	52	13 [25.00] + 2 SE	0
Total	758	267 [35.22] + 52 SE	44

*Single shoots or cluster of shoots.

**Plantlets developed from somatic embryos.

MS—Murashige & Skoog^38^ medium.

Somatic embryos (SE) were induced on shoot tips and cotyledons (Table 2, Fig. 1J) after explants transfer from inducing medium (MS + 0.25 or 0.50 mg L^−1^ BAP) onto ½ MS (2% saccharose); the 52 largest plants developed from SE (Fig. 1K), and 267 rooted shoots (Table 3) were moved to *ex vitro* conditions, transferred to pots filled with deacidified peat mixed with sand (Fig. 1L–N) and they grew first indoor and then under garden conditions till transfer to the field site.

### Genetic compatibility of regenerated plants with initial material

**Figure 2:**
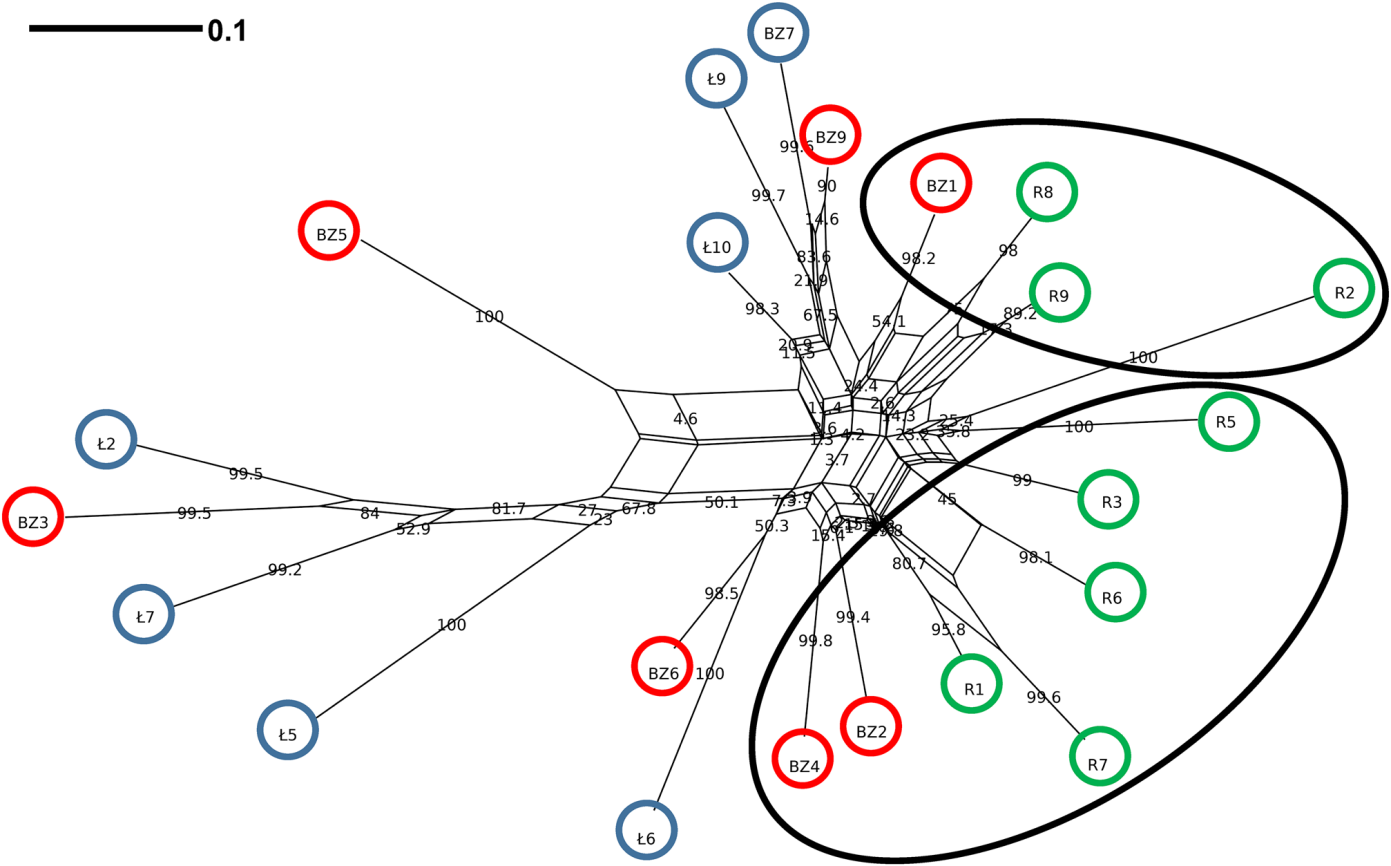
NeigborNet of *Pulsatilla patens* constructed on 51 ISSR loci using Dice distance. Branch support values are based on bootstrap analysis with 1000 replicates. Ł—plants from natural population in Łagiewniki near Busko-Zdrój, marked in blue circles; BZ—seedlings from seeds harvested in Łagiewniki near Busko-Zdrój, marked in red circles; R—regenerated plants from Łagiewniki near Busko-Zdrój seedling explants, marked in green circles.

NeighborNet analysis showed some diversity among plants from natural population in Łagiewniki near Busko-Zdrój (Ł), which clustered in two groups together with plants derived from the seeds from this population (BZ). The most divergent group (bootstrap 81.7) contained plants Ł2 and Ł7 along with BZ3 seedling derived from Ł2. Most of the other plants were grouped in large second cluster together with regenerated plants (R1–R9), which exhibited low divergence and non-significant grouping (branch support < 50) (Fig. 2). Seedlings likely originated from seeds of different initial plants: BZ3 from Ł2; BZ6 from Ł6; BZ9 and BZ7 from Ł9. Regenerated plantlets (R1–R9) were genetically close to initial plants BZ1, BZ2, BZ4 (Fig. 2). NeighborNet analysis showed non-significant genetic diversity between plants from southern (Ł, Łagiewniki near Busko-Zdrój) and north-eastern Poland (K; Kolimagi near Kolno), with the highest bootstrap value (45.4) between some of BZ, Ł and K plants (BZ3; Ł2, Ł7; K4, K5) and the rest of the samples (Suppl. Fig. 1).

Genome size for analyzed diploid *Pulsatilla* species (2*n* = 2*x* = 16) varied from 8.80 to 11.42 pg/2C (Table 4). *P. slavica* was a tetraploid with 2*n* = 4*x* = 32 and possessed the largest genome (25.13 pg/2C). For *P. patens*, mean genome size was 2C = 10.93 pg. Detected differences between regenerated plants and the initial material as well as plants from the natural population Łagiewniki near Busko-Zdrój, and two other locations were statistically non-significant. Genome size of plants from natural population in Łagiewniki near Busko-Zdrój (11.42 pg/2C) was higher than 2C-value established for the commercial cultivar from “Magda” Garden Center (10.85 pg/2C).

**Table 4:** Chromosome number and genome size (2C DNA) of different *Pulsatilla* species.

Taxon	Chromosome number 2*n*^1^	Material origin	2C DNA	
			Mean (pg)	SD±
*P. patens* (L.) Mill.	16^1^^,4^, 32^3^	Plants collected in Kolimagi near Kolno—Natura 2000 “Sasanki w Kolimagach”, NE Poland; N = 11	10.86	0.14
		Plants collected in Janów Lubelski—Natura 2000 „Uroczyska Lasów Janowskich”, SE Poland; N = 2	11.19	0.32
		Plants purchased from "Magda" Garden center; N = 16	10.85*	0.16
		Plants collected in Łagiewniki near Busko-Zdrój, SE Poland; N = 3	11.42*	0.08
		Plants obtained from seeds collected in Łagiewniki near Busko-Zdrój; N = 6	10.93	0.10
		Plants regenerated from shoot tips of plants obtained from seeds collected in Łagiewniki near Busko-Zdrój and plants converted from somatic embryos; N = 12	10.95	0.12
**Mean for the species (± SD)**			**10.93**^**a**^** (± 0.20)**	
*P. vernalis* L.	16^2^	Plants from the Forest Inspectorate Kaliska; N = 9	11.76^b^	0.15
*P. slavica* G. Reuss	32^1^	Plants obtained from seeds received fromTatra Field Station, Zakopane, S Poland; N = 7	25.13^c^	0.17
*P. alpina* (L.) Delarbre	16^5^	Plants collected on Kasprowy Wierch, the Tatra Mts., S Poland; N = 3	8.80^d^	0.16

Mean 2C DNA values for groups within *P. patens* marked with * differ significantly according to Kruskal–Wallis test. Mean 2C DNA values for each species marked with different letters (a–d) differ significantly according to one-way ANOVA and a Tukey's HSD *post-hoc* test for different N. SD–standard deviation.

^1^From^39^.

^2^From^40^.

^3^From Löve 1954 (cited from^40^).

^4^Present paper.

^5^From^41^.

### Reintroduction of plantlets

**Figure 3:**
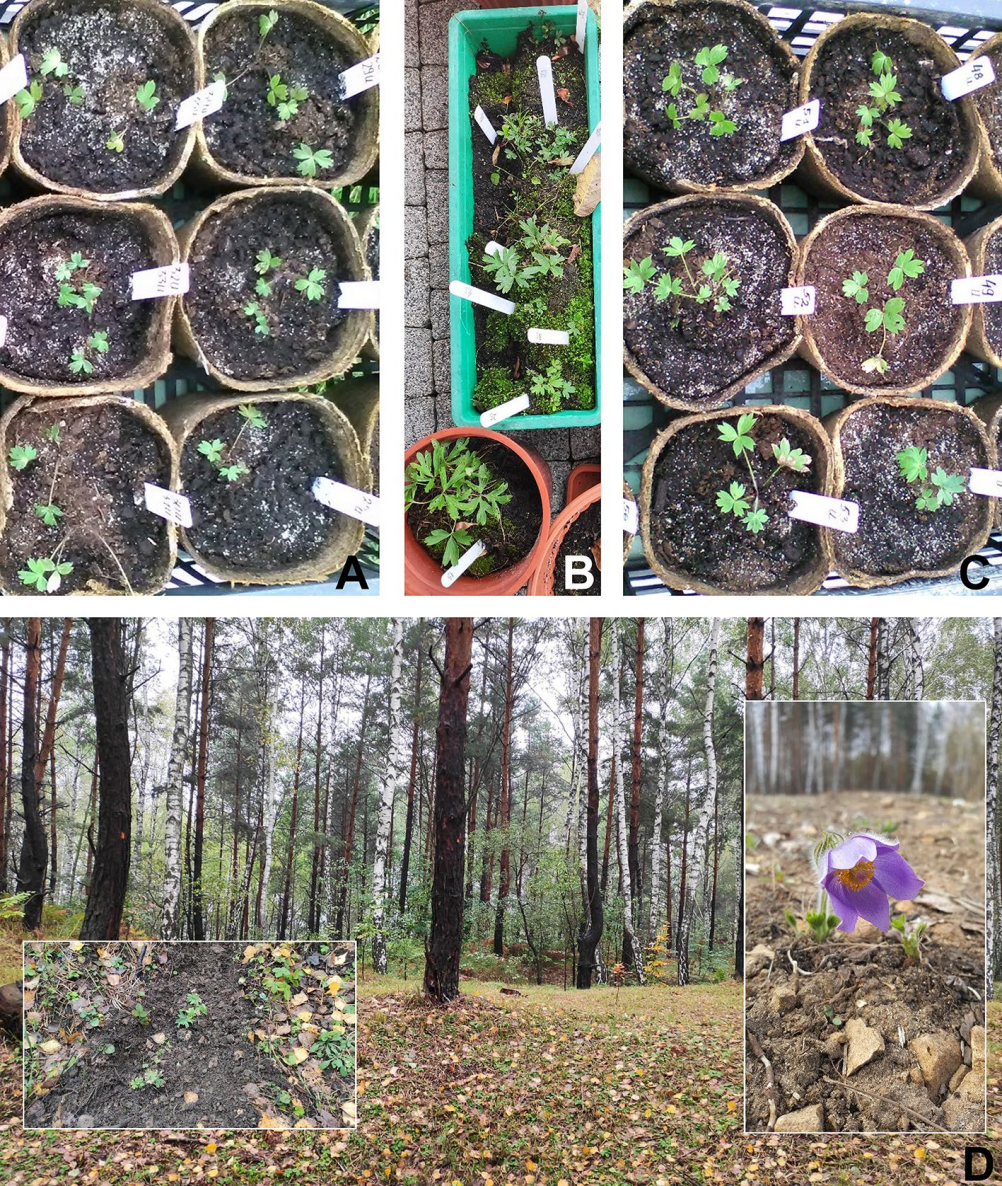
*Pulsatilla patens* plants used for revitalization of extinct population on Sadowa Góra in Jaworzno. (**A**, **B**) Plants from seeds from Łagiewniki near Busko-Zdrój natural population germinated in vitro on ½ MS (Murashige & Skoog) + 2% saccharose (**A**) and in substrate: garden soil (**B**). (**C**) Plants regenerated in vitro via organogenesis or somatic embryogenesis. At these stages of the development plants (**A**–**C**) were transplanted on Sadowa Góra in Jaworzno in October 2020. (**D**) Sadowa Góra (Jaworzno), open area at the edge of the forest; on the left transplanted plants in 2020 (inserted), on the right flowering plant in April 2021.

In total, 132 propagated plants, 97 from seeds (including 17 from Kolimagi near Kolno, NE Poland), 30 micropropagated in vitro, and five from rhizome cuttings, were transplanted to the field site in 15 October 2020 (Fig. 3A–D). Before transplantation, six plots were selected in this area with varying degrees of shade (Table 5), on which propagated plants were dug into. Out of 44 plants micropropagated in vitro, 30 were introduced to the field site, and the remaining 14, that were too small for introduction to the field, were stored under greenhouse conditions until the following season. In April 2021, the monitoring of all six plots on Sadowa Góra revealed that out of 132 transplanted *P. patens*, 40 (30.33%) had survived the first winter, and some developed flowers (Fig. 3D right insert) and set seeds (Table 5). In next season (March/April 2022) the number of surviving individuals decreased to 25.00% while the number of flowering and fruiting shoots in different clumps increased in some plots, even to over 20 (plots 2, 6; Table 5). Fruits developed well and were filled with seeds (Table 5).

**Table 5:** Success of reintroduction of *Pulsatilla patens* on Sadowa Góra in Jaworzno—rate of surviving plants in two subsequent seasons (March/April 2021/2022) after the reintroduction.

Plots for plants transplantation (October 2020)	No. of transplanted plants in 2020 and plant origin	Seasons 2021/2022			
		No. of plants surviving^a^	No. of plants at generative stage	No. of flowering shoots (in different clumps)	No. of fruiting shoots (in different clumps)
1. Slightly recessed area located at the top of a small mound formed after calamine mining; area completely exposed	**26** Nine from seeds germinated in vitro Two from seeds coll. in Kolimagi germinated in garden soil Six from seeds coll. in Łagiewniki germinated in garden soil Five from cutting rhizomes Four regenerated in vitro	5/5	4^a^/5	7^a^/17	6^a^/16
2. A periphery of the excavation with SE exposure and a slope of approx. 10°; area completely exposed	**15** Four from seeds germinated in vitro Four from seeds coll. in Łagiewniki germinated in garden soil Seven regenerated in vitro	10/9	3^b^/7	6^b^/27	6^b^/23
3. A periphery of the small excavation with NW exposure and an inclination of approx. 25°; area partially shaded	**8** One from seeds germinated in vitro One from seeds germinated in peat Two from seeds coll. in Łagiewniki germinated in garden soil Four regenerated in vitro	2/0	0/0	0/0	0/0
4. A periphery of the shallow excavation with exposure E and SE and an inclination of approx. 10°; area partially shaded by Scots pine trees growing in the vicinity	**35** Seven from seeds germinated in vitro Eight from seeds coll. in Kolimagi germinated in garden soil Nighteen from seeds coll. in Łagiewniki germinated in garden soil One regenerated in vitro	12/8 (4 clumps dug up by visitors)	3^b^/6	7/11	6/10
5. A periphery of the shallow with Scots pine in the center; SE exposure and a slope of approx. 15°; area partially shaded by Scots pine	**22** Five from seeds coll. in Kolimagi germinated in garden soil Twelve from seeds coll. in Łagiewniki germinated in garden soil Five regenerated in vitro	7/7	2^b^/6	3/16	2/11
6. A periphery of the shallow excavation with SE exposure and an inclination of approx. 25°; area completely exposed	**26** Two from seeds coll. in Kolimagi germinated in garden soil Fifteen from seeds coll. in Łagiewniki germinated in garden soil Nine regenerated in vitro	4/4	1^b^/4	1/20	1/19
Total	**132**	40 [30.33]/33 [25.00]	13/28	24/91	21/79

^a^Plants from cutting rhizomes or from two-year-old plants from seeds collected in Kolimagi (Natura 2000 “Sasanki w Kolimagach”, NE Poland).

^b^Two-year-old plants from seeds collected in Kolimagi.

## Discussion

The research objectives of this study were successfully achieved, including a completed life cycle of *P. patens* starting with sowing seeds into various substrates for plant multiplication, in vitro regeneration of plantlets, acclimatization of multiplied plants to the field conditions, re-introduction to the calamine grassland, and finally monitoring the frequency of plants that survived and developed flowers, fruits and set seeds in the following seasons. If the appropriate habitat is maintained in the following years, the recovered extinct population will contribute to the preservation of biodiversity of calamine grasslands^42^.

### Preservation of the biodiversity of calamine grasslands with particular emphasis on pasque-flower

Recently, the discussion concerning the value of natural areas associated with the extraction and processing of Zn–Pb ores has been focused on the requirement of active protection of this unique community^43^. Under the EU Habitats Directive Annex I (Fauna-Flora-Habitat), heavy-metal vegetation is coded as calamine grasslands (6130). On the list of Sites of Community Importance, two Natura 2000 areas protecting calamine grasslands in Polish Silesian-Cracow region (“Pleszczotka”—PLH120092 and “Armeria”—PLH120091) are included. The presented research carried out within the project: “Good practices for enhancing biodiversity and active protection of calamine grasslands in the Silesia-Cracow region BioGalmany”, co-financed by the EU under the ESF, POIiŚ 2014–2020, POIS.02.04.00-00-0043/17-0, are a part of broad initiative to protect calamine grasslands.

Important factors influencing the decline in the number of individuals of that species, leading to the population decline and disappearance thorough Europe are poor seed germination and scarce seedling survival. An adult plant produces from one to several dozen of self-compatible and protogynous flowers^44^ that are pollinated by insects, mainly bees^45^. As the separation of the male and female sexual phases in the flower is not complete, self-pollination might occur, however, very rarely^44^. The production of fruits/seeds is high, since each flower might produce on average > 100 monospermed achenes^17^. Seeds retain viability and germination capacity for several years, thus forming a transient seed bank^7^. Hence the factors limiting germination and seedling development are the exact timing in late autumn the first year or early spring of the following year; critical factors are humidity and low temperatures and the thickness and compactness of the moss cover and the amount of accumulated litter^46^. For the persistence of the population it is necessary to maintain a relatively large diversity of microhabitats, that reduce seedling mortality, e.g. due to desiccation^9,17^. These factors were considered when preparing the area on Sadowa Góra for the re-introduction of *P. patens* multiplied plants. Six plots were selected with different degrees of canopy openness and slope within a historical forest, where some trees had been earlier removed (Table 5).

The survival rate of plants introduced to six plots on Sadowa Góra seems to be dependent on several factors. The most important is the developmental stage of introduced plants—juvenile with 2–3 leaves vs larger with several well-developed leaves. The survived individuals were introduced as one- or two-year-old plants originated from seeds or rhizome cuttings which allow us to conclude that the multiplied plant material must be grown at least two seasons in the field/experimental garden conditions before the introduction. The positive correlation between the insolation of the site and clumps growth/generative organ development was noticed. At completely exposed plots no. 2 and 6, not shaded by Scots pine, the clumps expanded and developed even dozens flowering/fruiting branches (Table 5). In open space pollinator access to flowers must have been easier as evidenced by the development of fruits at these sites.

### Good practices of introducing pasque-flower fulfilled

Several *Pulsatilla* species, e.g. *P. grandis*, *P. koreana*^47^, *P. patens*^12,47^, *P. pratensis*^48–50^, *P. turczaninovii*^51^*, P. vernalis* and *P. vulgaris*^33^, were multiplied by in vitro organogenesis and/or somatic embryogenesis, acclimatized to *ex vitro* and to semi-natural conditions. However, in none of these experiments, regenerated plants were re-introduced to the natural (extinct) populations, and their genetic uniformity was not confirmed with the initial material. It is well-known that in vitro conditions generate genome alteration (gene/genome mutations, epigenetic changes) of regenerated plants leading to the formation of new genotypes (e.g. aneuploids, polyploids). The introduction of such somaclonal variants into natural populations could disrupt the genetic structure of the population and even lead to the elimination of existing genotypes, as discussed by Żabicki et al.^31^. The genetic diversity of in vitro regenerated *P. patens* plants estimated in our research by ISSR molecular markers fell within the range of natural populations of this species. Plants multiplied in vitro and introduced into the field did not differ genetically from the initial seedlings and can be treated as one line (genotype). All regenerants represented the same ploidy level/genome size as the initial material and individuals from natural populations.

Our study concerned the re-introduction of the extinct population on Sadowa Góra (S Poland), where in the past *P. patens* was common in a large calamine grassland. Plants used for introduction were multiplied from the initial material originated from another (not adjacent) region of S Poland. We had herbarium specimens from the calamine grasslands, unfortunately, the quality of the isolated DNA was insufficient for ISSR analysis to show genetic uniformity of the multiplied material with the calamine extinct population. Based on low genetic differentiation of *P. patens* found in different areas of its range indicating higher levels of variation within populations than between populations^13^, we assumed that the genotypes obtained via multiplication of the material from S Poland population Łagiewniki near Busko-Zdrój is not genetically different from the extinct population on Sadowa Góra. To confirm this, we studied individuals from north-eastern Poland (Kolimagi near Kolno) using ISSR analysis. The results showed that there was no significant genetic difference between plants from Łagiewniki near Busko-Zdrój and Kolimagi near Kolno plants, and the rest of the samples, including regenerants. Also, two-year-old plants obtained from seeds collected at Kolimagi near Kolno and transplanted to Sadowa Góra were well adapted to their climatic and habitat conditions. This suggests that we did not introduce aberrant genotypes of *P. patens* into the place of the extinct population. The transplanted plants reproduced sexually producing fully viable pollen and set seeds.

### Future perspectives

Micropropagation protocol of *P. patens* developed in our studies makes it possible to preserve the genetic pool of an endangered species by cryopreservation. The next step in conservation practice is developing an effective procedure for cryopreservation of shoot tips, shoots or somatic embryos of *P. patens* and introducing them for long-term storage in liquid nitrogen, including individuals from different European populations. Although several rare, endemic and endangered species have already been introduced to cryogenic conditions^32,52–54^, thousands still need to be cryopreserved, including all *Pulsatilla* species. It should be emphasized that in situ protection alone of endangered species will not guarantee their conservation, and thus must be supported by ex situ management with the use of biotechnological techniques and optionally seed banking as complementary for plant conservation including short-, medium- and long-term strategies^54^.

## Conclusions

The breakthrough of this study is successful multiplication of the endangered species *P. patens* using different techniques, including in vitro regeneration via organogenesis and somatic embryogenesis, acclimatization to the field conditions of multiplied plants and their transplantation into the nature. Our studies could serve as a model for restoring extinct populations and preserving the biodiversity of rare species.

## Material and methods

### Plant material

The *Pulsatilla* material used in this study is described in Table 6. As all *Pulsatilla* species used are under protection in Poland, the materials were collected with the permissions of the Regional Directorate for Environmental Protection (RDEP). We did not obtain the permission from RDEP to collect for an herbarium, because it would be a direct and poorly justified destruction of plants. The species were identified by plant taxonomists (see footnotes under Table 6, Suppl. Fig. 2). Experimental research on this wild plant, including the collection of plant material, complied with relevant institutional, national, and international guidelines and legislation.

**Table 6:** *Pulsatilla* species used for different analyses.

Material	Locality	Analysis/techniques
Ripe fruits of *P. patens*	Natural population in Łagiewniki near Busko-Zdrój (SE Poland)	Seed germination frequency; seedlings development; explants for in vitro culture; callus for histological analysis, chromosome counting
Flowers/anthers of *P. patens*	Natural population in Łagiewniki near Busko-Zdrój (SE Poland); propagated plants transplanted on Sadowa Góra in Jaworzno (SW Poland)	Pollen grains stainability/viability test
Clumps of *P. patens*	Natural population in Łagiewniki near Busko-Zdrój (SE Poland)	Cutting rhizomes for vegetative propagation
**Leaves of *P. patens* and other *Pulsatilla* species**		
*P. patens* plants^1^; seedlings; in vitro regenerated plants	Natural population in Łagiewniki Busko-Zdrój (SE Poland); Kolimagi near Kolno, Natura 2000 „Sasanki w Kolimagach” (NE Poland)	Genome size by flow cytometry; ISSR markers for genetic differentiation
*P. patens*^2^	Janów Lubelski, Natura 2000 „Uroczyska Lasów Janowskich” (SE Poland); cultivar from garden center "Magda" in Cracow (S Poland)	Genome size by flow cytometry
*P. vernalis*^3^	Forest Inspectorate Kaliska (N Poland)	
*P. slavica*^4^	Center for Research and Protection of Mountain Plants of the Institute of Nature Conservation of the Polish Academy of Sciences, Tatra Field Station, Zakopane (S Poland)	
*P. alpina*^5^	Kasprowy Wierch, Tatra Mts. (S Poland)	

^1^^,2^—Dr. Monika Jędrzejczyk-Korycińska & dr. Teresa Nowak from the University of Silesia in Katowice (Katowice, Poland).

^3^—Employees of the Forest Inspectorate Kaliska.

^4^—Prof. Szymon Zubek from the Jagiellonian University in Kraków (Cracow, Poland).

^5^—Prof. Zbigniew Mirek from Polish Academy of Science (Cracow, Poland).

### Chromosome counting and pollen grain stainability/viability test

Roots of *P. patens* seedlings developed (on wet cellucotton) from seeds harvested in Łagiewniki near Busko-Zdrój (Ostoja Szaniecko-Solecka” PLH260034) were pretreated in a saturated solution of alpha-bromonaphthalene (Sigma-Aldrich, USA) at room temperature for 4 h, then fixed in a mixture of 96% ethanol and glacial acetic acid (3:1, v/v) for 24 h, stained with 2% solution of orcein (Fluka, Switzerland) for 72 h. Dissected root tips were squashed in 45% acetic acid and covered with coverslips. Chromosome numbers were determined on four selected metaphase plates.

Anthers from randomly collected five, fully opened deep violet flowers of *P. patens* (Fig. 1A) from Łagiewniki near Busko-Zdrój and from transplanted plants to Sadowa Góra were kept in 70% ethanol until use. Pollen grains (300 from each site) were stained using Alexander dye^55^. Viable pollen stained purple-red, non-viable green. Chromosomes and pollen grains were photographed under Nikon Eclipse E400 light microscope equipped with camera and NIS-Elements Viewer imaging software ver. 4.00 (Tokyo, Japan).

### Seed germination test, seedlings development, vegetative propagation

Mature fruits with seeds of *P. patens,* collected randomly from at least 15 plants in Łagiewniki near Busko-Zdrój, were germinated on different substrates, i.e. under sterile in vitro conditions on Petri dishes filled in with ½ MS^38^ medium with 2% saccharose solidified with 0.8% agar (w/v) (pH 5.7–5.8), in garden soil (pH 6.0–6.5) or in deacidified peat (pH 5.5–6.5) mixed with sand (3:1 v/v) in pots. Fruits harvested (at the same manner as in Łagiewniki near Kolno Natura 2000 site „Sasanki w Kolimagach” PLH200025, NE Poland) were germinated in garden soil (pH 6.0–6.5). Fruit pappus-hairs were removed from each fruit with scissors or tweezers. For in vitro germination, fruits were sterilized according to Żabicka et al.^33^ without any other pretreatment. Germination procedure was performed in triplicate.

Seedlings obtained from seeds germinated in vitro on ½ MS supplemented with 2% saccharose and transplanted to pots filled with peat mixed with sand (3:1 v/v) or germinated directly in garden soil in pots were acclimatized. Depending on the time of sowing the seeds, sprouting was directly in outdoor garden conditions (in spring), or first the seedlings were acclimatized indoors several weeks/months (late summer–winter) and then, in spring, transferred to the garden. Acclimatized seedlings were introduced on Sadowa Góra in Jaworzno in October 2020. At the time of planting on Sadowa Góra, the plants were one- or two-year-old.

Two mature patches of *P. patens* that had been dug up in the natural population Kolno Natura 2000 “Sasanki w Kolimagach” in June 2018 were transferred to garden conditions. In late November, when plants were dormant, their rhizomes were divided into several parts, moved to pots with garden soil and left till following season. Vegetatively obtained new plants based on rhizome cuttings were introduced on Sadowa Góra at the same time as acclimatized plants obtained from seeds.

### Media and in vitro culture conditions

The explants, cotyledons, hypocotyls and roots, shoot tips obtained from 4–5-week old seedlings were transferred on MS supplemented with 0.25 or 0.5 mg L^−1^ BAP (Benzylaminopurine) inducing media solidified with 0.8% agar (w/v) according to Priede and Kļaviņa^56^. The explants were passaged onto the same fresh media every 4 or 6 weeks, then adventitious shoots or clusters of adventitious shoots were transferred onto ½ MS (with 2% saccharose) rooting medium. Cultures were maintained in a growth chamber at 25 ± 3 °C under a 16 h photoperiod (cool-white fluorescent lamps, 60–90 μmol m^−2^ s^−1^); the experiment was repeated in triplicate.

### Histological technique

To document organogenesis and somatic embryogenesis, proliferated on explants callus was fixed in the mixture of glacial acetic acid and 96% ethanol (1/3, v/v), dehydrated in an ethanol series, embedded in paraffin, sectioned in 10–12 μm slices on a rotary microtome (Adamas Instrumenten BV, HM 340E, Netherlands), stained with Ehrlich’s hematoxylin (Fluka, Switzerland) combined with alcian blue (Fluka, Switzerland). Stained sections were mounted in Entellan (Sigma-Aldrich, USA). The histological sections were analyzed and photographed under the same microscope as chromosomes and pollen grains.

### Genome size estimation

Leaves of different *Pulsatilla* species, of plants obtained from seeds collected in Łagiewniki near Busko-Zdrój, from adventitious shoots or somatic embryos regenerated in vitro, from Kolimagi near Kolno (Natura 2000 „Sasanki w Kolimagach”, NE Poland), Janów Lubelski (Natura 2000 „Uroczyska Lasów Janowskich”, PLH060031, SE Poland), and of a cultivar from garden center "Magda" in Cracow (S Poland) (Table 6) were analyzed by flow cytometry (FCM). Apart from *P. patens*, in the genome size research, we included other pasque-flower species found in Poland, which was dictated by the fact that all pasque-flowers are either diploids (2*n* = 16) or tetraploids (2*n* = 32). Since chromosome numbers alone are inconclusive in species designation, we decided to measure their genome size to uniquely identify the material used in the research. *Secale cereale* (2C = 16.19 pg)^57^ served as an internal standard. For nuclei isolation the buffer developed by Marie and Brown^58^ was used, and for DNA staining propidium iodide (PI, 50 μg mL^−1^; for details of sample preparation see^59^. Nuclear DNA content in at least 5000 nuclei per sample was estimated using a CyFlow SL Green (Partec GmbH, Münster, Germany) flow cytometer. Histograms were evaluated using a FlowMax program (Partec GmbH).

The coefficient of variation (CV) of the G_0_/G_1_ peak of *Pulsatilla* ranged from 2.51 to 4.68%. Nuclear DNA content was calculated using the linear relationship between the ratio of the 2C peak positions of *Pulsatilla*/*Secale* on a histogram of fluorescence intensities. The number of biological replications varied from 2 to 16. Low number of analyzed plants of some entries from natural sites was due to the restrictions of the regional Directorate for Environmental Protection or a small number of individuals in the population.

### ISSR markers for genetic differentiation

Leaves dried in silica gel (silicon dioxide; F.H.U. “DOR-CHEM”, Poland) of plants from Kolimagi near Kolno (Natura 2000 “Sasanki w Kolimagach”, PLH200025, NE Poland), Łagiewniki near Busko-Zdrój, plants obtained from seeds, and plants regenerated from shoot tips of seedlings (Table 1) were used for molecular analysis. CTAB extraction method^60^ was used for DNA isolation. Quality of DNA was checked on 1% agarose gel. The analysis of genetic diversity using five primers of ISSR markers (Suppl. Table 1)^61,62^, and electrophoresis were based on protocols developed by Żabicka et al.^33^. Selected samples were used for test of repeatability of results. Results of analysis using ISSR markers of 27 samples, were captured with a MultiDoc-It™ Imaging System with VisionWorks LS Analysis Software (UVP, Upland, CA, USA, https://www.labortechnik.com/en/vision-works-ls/analysis-software). SplitsTree v. 4.6^63^ was used for splitting phylogenetic network (NeighborNet) construction, based on Dice coefficient. Bootstrap was calculated on 1000 replicates.

### Acclimatization to field conditions and transplantation to the place of extinct population in Southern Poland

*Pulsatilla patens* plants propagated from seeds, rhizome cuttings or micropropagated in vitro were gradually acclimatized to outdoor conditions (transferred from indoor to garden conditions) in the 2019–2020 seasons. In October 2020, all plantlets with several well-developed leaves were transplanted to nature and landscape complex Sadowa Góra (Jaworzno, Southern Poland), where in the past (till the 1980s) *P. patens* formed a large population in calamine grassland. Here, six site types (plots) were used: (1) slightly recessed area located at the top of a small mound formed after calamine mining—area completely exposed; (2) adjacent to the excavation with SE exposure and a slope of approx. 10°; area completely exposed; (3) nearby the small excavation with NW exposure and an inclination of approx. 25°—area partially shaded; (4) next to the shallow excavation with exposure E and SE, and an inclination of approx. 10°—area partially shaded by Scots pine trees growing in the vicinity; (5) next to the shallow excavation with Scots pine in the centre—SE exposure and a slope of approx. 15°, and area partially shaded by Scots pine; and (6) next to the shallow excavation with SE exposure and an inclination of approx. 25°—area completely exposed.

Plants were introduced to the field under the permission of the mayor of the city Jaworzno within the project “Good practices for enhancing biodiversity and active protection of calamine grasslands in the Silesia-Cracow region BioGalmany”. The site monitoring and the observation of introduced plants (number of survived individuals/clumps, flowering and fruiting of shoots in different clumps) were conducted in two subsequent seasons (March/April 2021/2022).

### Statistics

To evaluate the statistical significance of media composition/explant type impact on the in vitro culture results the two-way ANOVA followed by Tukey's HSD *post -hoc* test (P = 0.05) was used; 2C DNA genome size differences in a group of *P. patens* or between *Pulsatilla* species were performed using Kruskal–Wallis test or one-way ANOVA and a Tukey's HSD *post -hoc* for different N, respectively in Statistica 13.3 software (TIBCO Software Inc., Palo Alto, California, USA).

## Supplementary Information

Supplementary Figure 1.

Supplementary Figure 2.

Supplementary Table 1.

## Data Availability

The datasets generated during the current study are available in the Jagiellonian University Repository, DOIs: 10.26106/e0zr-gx31 (https://ruj.uj.edu.pl/xmlui/handle/item/289392), 10.26106/7sn7-hy05 (https://ruj.uj.edu.pl/xmlui/handle/item/289393).
