# Supplementary material for: Re-introduction of an extinct population of Pulsatilla patens using different propagation techniques
Source: Sci Rep. 2022 Aug 22;12:14321. doi: 10.1038/s41598-022-18397-0 (PMC9395332; doi:10.1038/s41598-022-18397-0)
Supplement: Supplementary file 1 — Supplementary Figure 1. [file 41598_2022_18397_MOESM1_ESM.pptx]

## Slide 1
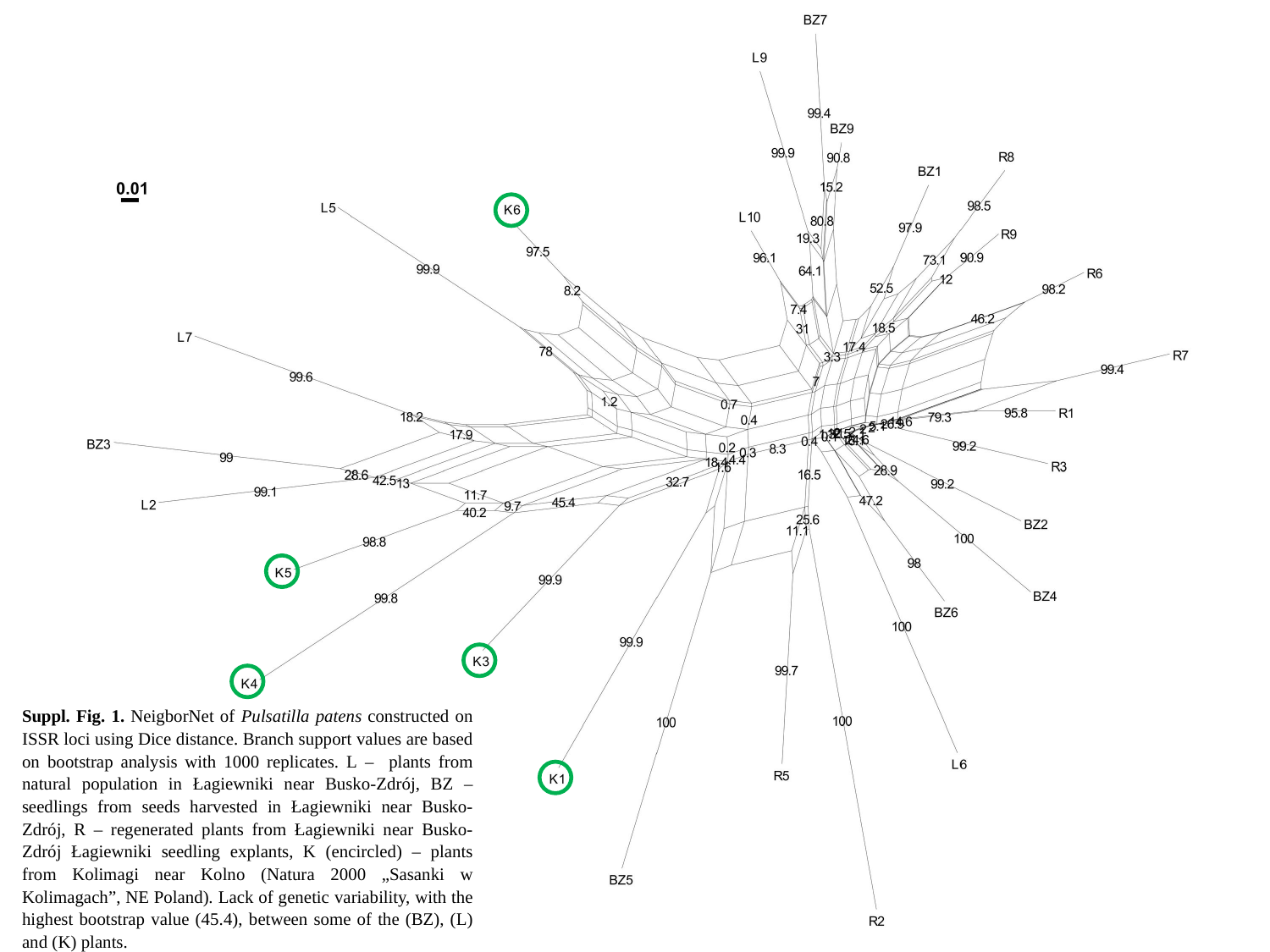

#
Suppl. Fig. 1. NeigborNet of Pulsatilla patens constructed on ISSR loci using Dice distance. Branch support values are based on bootstrap analysis with 1000 replicates. L – plants from natural population in Łagiewniki near Busko-Zdrój, BZ – seedlings from seeds harvested in Łagiewniki near Busko-Zdrój, R – regenerated plants from Łagiewniki near Busko-Zdrój Łagiewniki seedling explants, K (encircled) – plants from Kolimagi near Kolno (Natura 2000 „Sasanki w Kolimagach”, NE Poland). Lack of genetic variability, with the highest bootstrap value (45.4), between some of the (BZ), (L) and (K) plants.
