# Supplementary material for: Re-introduction of an extinct population of Pulsatilla patens using different propagation techniques
Source: Sci Rep. 2022 Aug 22;12:14321. doi: 10.1038/s41598-022-18397-0 (PMC9395332; doi:10.1038/s41598-022-18397-0)
Supplement: Supplementary file 2 — Supplementary Figure 2. [file 41598_2022_18397_MOESM2_ESM.docx]

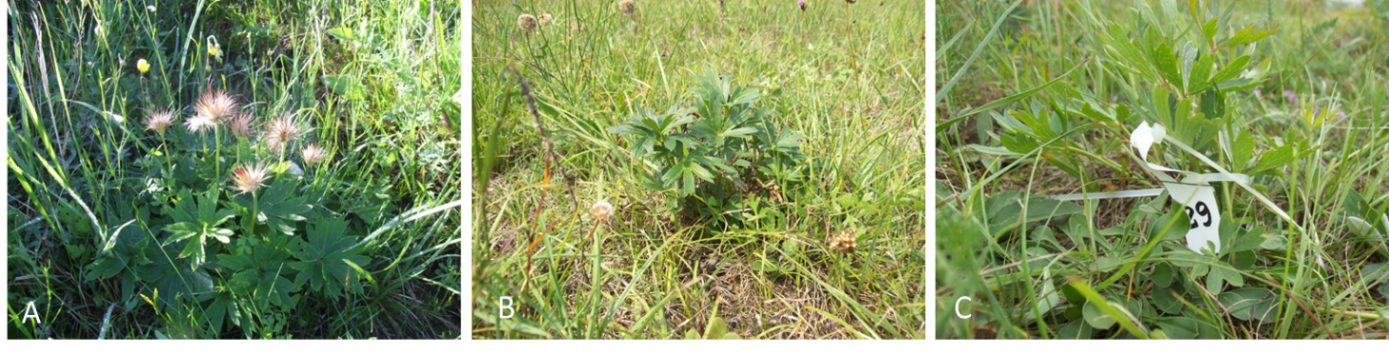
**Suppl. Fig. 2.** *Pulsatilla patens* specimens growing in natural populations (2019 and 2020): Łagiewniki near Busko-Zdrój (S Poland) (A), Kolimagi near Kolno (NE Poland) (B, C). Photos by T. Nowak.
