# Supplementary material for: Re-introduction of an extinct population of Pulsatilla patens using different propagation techniques
Source: Sci Rep. 2022 Aug 22;12:14321. doi: 10.1038/s41598-022-18397-0 (PMC9395332; doi:10.1038/s41598-022-18397-0)
Supplement: Supplementary file 3 — Supplementary Table 1. [file 41598_2022_18397_MOESM3_ESM.docx]

**Suppl. Table 1.** ISSR primers and their annealing temperatures.

| Primer | Primer sequence (5'-3') | Annealing temperature (°C) |
| --- | --- | --- |
| ISSR1 | 5'-TCTCTCTCTCTCTCTCC-3' | 50.2 |
| ISSR2 | 5'-AGAGAGAGAGAGAGAGT-3' | 49.9 |
| ISSR3 | 5'-ACACACACACACACACYC-3' | 50.3 |
| ISSR4 | 5'-GACAGACAGACAGACA-3' | 44 |
| ISSR5 | 5'-ACTGACTGACTGACTG-3' | 44 |
